# Latent Epstein-Barr Virus Can Inhibit Apoptosis in B Cells by Blocking the Induction of NOXA Expression

**DOI:** 10.1371/journal.pone.0028506

**Published:** 2011-12-09

**Authors:** Jade Yee, Robert E. White, Emma Anderton, Martin J. Allday

**Affiliations:** Section of Virology, Division of Infectious Diseases, Faculty of Medicine, Imperial College London, London, United Kingdom; Karolinska Institutet, Sweden

## Abstract

Latent Epstein-Barr virus (EBV) has been shown to protect Burkitt's lymphoma-derived B cells from apoptosis induced by agents that cause damage to DNA, in the context of mutant p53. This protection requires expression of the latency-associated nuclear proteins EBNA3A and EBNA3C and correlates with their ability to cooperate in the repression of the gene encoding the pro-apoptotic, BH3-only protein BIM. Here we confirm that latent EBV in B cells also inhibits apoptosis induced by two other agents – ionomycin and staurosporine – and show that these act by a distinct pathway that involves a p53-independent increase in expression of another pro-apoptotic, BH3-only protein, NOXA. Analyses employing a variety of B cells infected with naturally occurring EBV or B95.8 EBV-BAC recombinant mutants indicated that the block to NOXA induction does not depend on the well-characterized viral latency-associated genes (*EBNAs 1*, *2*, *3A*, *3B*, *3C*, the *LMPs* or the *EBERs*) or expression of BIM. Regulation of NOXA was shown to be at least partly at the level of mRNA and the requirement for NOXA to induce cell death in this context was demonstrated by *NOXA*-specific shRNA-mediated depletion experiments. Although recombinant EBV with a deletion removing the *BHRF1* locus – that encodes the BCL2-homologue BHRF1 and three microRNAs – partially abrogates protection against ionomycin and staurosporine, the deletion has no effect on the EBV-mediated block to NOXA accumulation.

## Introduction

*In vitro*, EBV can very efficiently induce the activation and continuous proliferation of primary human B cells. The resulting ‘immortalized’ lymphoblastoid cell lines (LCLs) carry the viral genome as extra-chromosomal episomes and express only nine latency-associated EBV proteins. There are six nuclear antigens (EBNAs 1, 2, 3A, 3B, 3C & -LP), three membrane associated proteins (LMP1, LMP2A & 2B) and several untranslated RNA species (EBERs and BARTs). This programme of viral gene expression is known as latency III or the ‘growth programme’ [1], [2], [3].

However, data on the persistence of EBV in humans are consistent with the viral genome residing long-term in a resting memory B cell population in which no EBV proteins are expressed. It is now considered probable that to establish persistence, EBV initially infects resting (naïve) B cells and drives these to proliferate as activated B-blasts. This expanding infected B-blast population then migrates into germinal centres, where the infected cells differentiate to become centroblasts, centrocytes and finally resting memory B cells. While the precise series of events that the EBV-positive B cells undergo to reach the memory compartment is unknown, it appears to involve regulated shut-down of latency-associated viral gene expression from an initial state (latency III, as found in LCLs) in B-blasts, via latency II (EBNA1, the LMPs, the EBERs and BARTs) in centroblasts and centroctyes until in quiescent memory B cells no EBV protein transcripts can be detected in a state called latency 0. When resting memory cells re-enter the cell cycle and divide, EBNA1 alone becomes detectable (termed latency I) [3], [4], [5].

Although persistent infection is normally asymptomatic, EBV can be the causative agent in the benign lymphoproliferation known as infectious mononucleosis, and the virus is also etiologically linked to a number of human cancers including Burkitt's lymphoma (BL), Hodgkin's lymphoma, diffuse large B cell lymphoma, and epithelial-derived nasopharyngeal carcinoma [1], [6]. In normal individuals the accumulation of proliferating B cells is counterbalanced by differentiation into memory and also the action of EBV-specific cytotoxic T lymphocytes that recognise and destroy the proliferating B-blasts [7].

There is a great deal of evidence that EBV gene expression can very effectively suppress the process of programmed cell suicide known as apoptosis [8], [9], [10], [11], [12], [13], [14], [15], [16]. This ability of a virus to regulate apoptosis is generally considered to be important because apoptosis is a major antiviral response used by multicellular organisms for the removal of infected cells. EBV has therefore evolved multiple mechanisms to ensure the survival of infected cells long enough for the virus to establish persistent latent infection and subsequently undergo ‘lytic’ replication to produce new infectious virus. Furthermore, B cells – the primary target for EBV infection – are particularly prone to apoptosis when they pass through germinal centres because this is where cells that are not positively selected by high-affinity binding of antigen are removed. During B cell development, therefore, multiple regulators of apoptosis act to cull B cells at different stages in order to maintain homeostasis of the B cell compartment [17]. Inhibition of apoptosis by EBV is also generally considered to make a major contribution to the malignant transformation of infected cells, and hence the development of EBV-associated cancers – reviewed in [18], [19].

Numerous studies of EBV and apoptosis have identified most of the viral latency-associated gene products as having some anti-apoptotic activity (reviewed in [18], [19]), and a great deal of effort has gone into showing that the presence of latent EBV in BL-derived cells rescues them from apoptosis triggered by a wide range of exogenous stimuli – including cytotoxic drugs, cytokine deprivation, anti-IgM andγ-irradiation [13], [20], [21], [22]. This means comparison between studies is often difficult. For instance, we recently showed that the EBV-mediated protection against apoptosis specifically induced by either the DNA cross-linking agent cisplatin, the cyclin-dependent kinase inhibitor roscovitine or the mitotic spindle poison nocodazole is dependent on the co-expression of EBV latency-associated proteins EBNA3A and EBNA3C [21], [22]. This correlates with the down-regulation of the pro-apoptotic BH3-only protein BIM, which depends on expression of both EBNA3A and EBNA3C. This protection is independent of the tumour suppressor p53, which is often mutated in BL cells, including BL31 used in the study. However, we noted that when similar experiments were repeated using BL2 cells and their EBV-infected counterparts, both of which carry wild type p53 but fail to express BIM, latent EBV was unable to protect against apoptosis induced by the same agents [22]. This is because these genotoxic agents induce the stabilization and activation of the wild type p53 present in BL2 and the activation of an apoptotic pathway that is independent of BIM. Consistent with this, EBV does not block p53-induced apoptosis after DNA-damage to LCLs [23], [24].

In contrast with these observations, Kelly and colleagues found that latent EBV protects both BL31 cells (BIM-positive and carrying a mutant p53) and BL2 cells (BIM-negative and carrying wild type p53) equally well against apoptosis induced by the calcium-ionophore ionomycin and that the EBNA3 proteins appeared to play no role in this [20], [25]. We hypothesised that this must depend on a second pathway by which BL cells can be triggered to undergo apoptosis, that this is independent of EBNA3-, BIM- and p53-status, but can also be blocked very effectively by EBV. Here we have explored this alternative pathway and established that it involves the p53-independent accumulation of another pro-apoptotic, BH3-only protein, NOXA. The mechanism by which EBV protects the cells from this agent does not involve any of the well-characterised latency factors (including EBNA3A and EBNA3C), but is partly dependent on products of the EBV *BHRF1* locus – apparently acting alongside, but independently of NOXA.

## Results

### Confirmation that EBV latency-associated gene expression confers resistance against ionomycin-induced apoptosis

**Figure 1:**
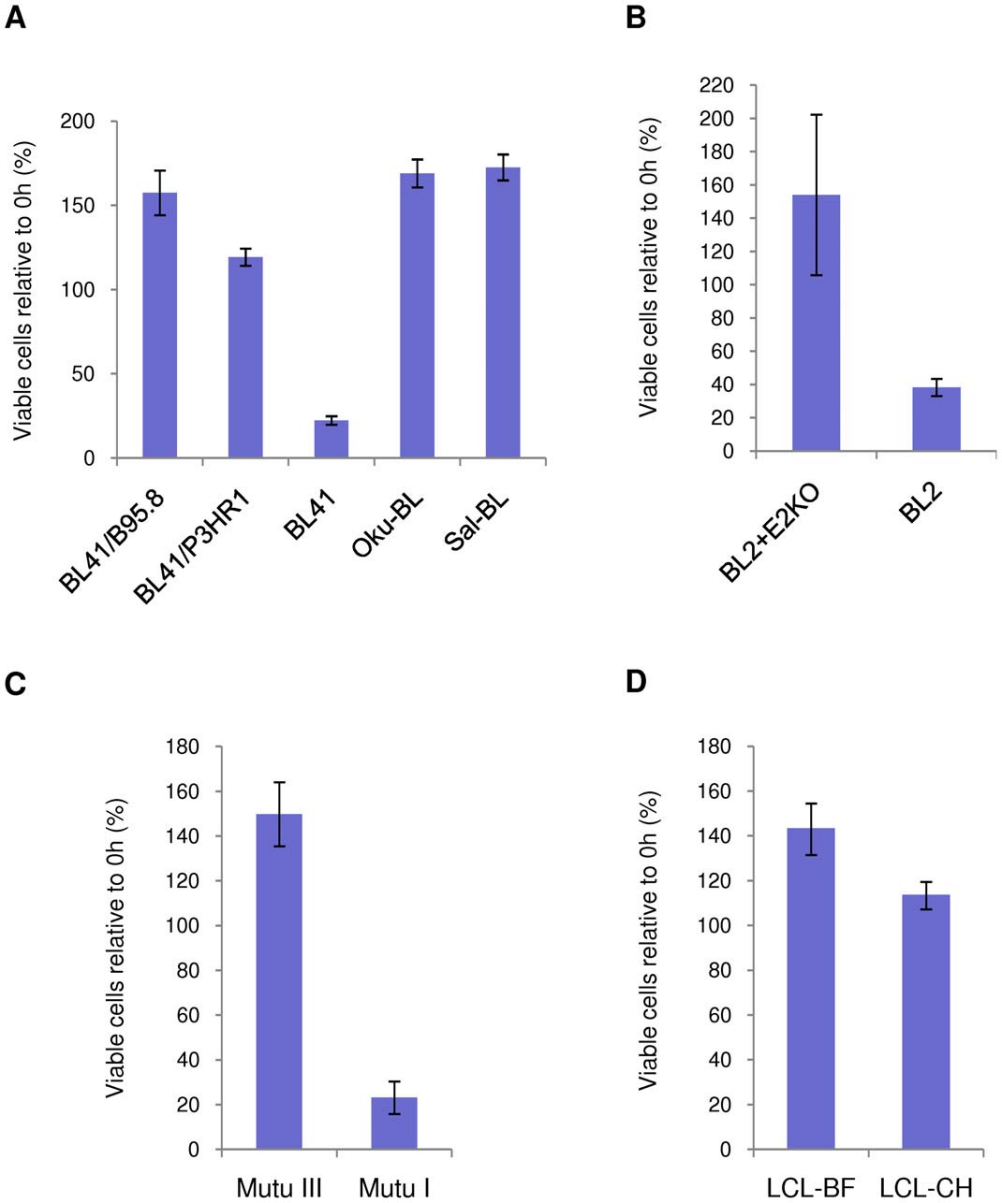
Latent EBV inhibits ionomycin-induced apoptosis in a variety of cell backgrounds. (**A**) BL41 and its EBV-positive counterparts, BL41/B95.8 and BL41/P3HR1, and two Wp-restricted BL Oku-BL and Sal-BL, (**B**) BL2 and its EBV-positive convert established with a recombinant EBNA2-knockout virus (BL2+E2KO), (**C**) Mutu III (expressing all the latency-associated proteins) and Mutu I (expressing only EBNA1) and (**D**) two LCLs were all treated with 1 µg/ml ionomycin (IM) for up to 48 hours. Viable cells (assessed by trypan blue exclusion) were counted. Cell survival is expressed as a percentage relative to the starting population and the mean and standard deviation of viable cell counts from three independent experiments are shown.

EBV-negative BL41 and two EBV-positive BL41 cell lines converted by infection using different virus strains, B95.8 and P3HR1, were treated with ionomycin at a concentration of 1 µg/ml for 48 hours (Figure 1A). BL41 is null for (WT) p53 because it carries only a single, mutant allele [26]. After treatment for 48 hours, fewer than 20% of BL41 cells remain viable. In contrast, BL41/B95.8 cells, which display the latency III phenotype, appear to be completely resistant to ionomycin-induced apoptosis and continue to proliferate. A similar response was observed in BL41/P3HR1 cells, which express a restricted set of latent proteins, as a result of a deletion in the region encoding EBNA2 and the two unique C-terminal exons of EBNA-LP (reviewed in [27]). As a result BL41/P3HR1 cells only express the latent proteins EBNA1, EBNA3A, EBNA3B, EBNA3C, a truncated form of EBNA-LP, and the EBERs and BARTs; the LMPs are not expressed because generally EBNA2 is required to transactivate their expression.

Similarly, the responses of more recently described BL lines, Oku-BL and Sal-BL, were also analysed (Figure 1A). These early passage cell lines were established from BL biopsy material [28] and found to carry EBV genomes with EBNA2 deletions similar to that of the P3HR1 strain. These cell lines are termed ‘Wp-restricted’ because the EBNA2 deletion causes the Wp promoter, rather than Cp, to remain the main originator of EBNA transcripts. Oku-BL and Sal-BL therefore express the same subset of EBV genes as BL41/P3HR1. However unlike BL41, both Oku-BL and Sal-BL carry wild-type (WT) p53 [22]. The data confirm that the ionomycin-resistant phenotype is not specific to BL41 and suggest that the restricted set of EBV proteins associated with the P3HR1 strain is sufficient to protect BL cells from ionomycin-induced apoptosis irrespective of the p53-status of cells.

BL2 cells – which have WT p53, but fail to express the pro-apoptotic factor BIM [22] – were infected with a recombinant EBV-BAC from which the EBNA2 open reading frame (ORF) had been deleted to produce a P3HR1-like virus (E2KO), but carrying a full length EBNA-LP [20]. As seen with EBV-negative BL41, BL2 cells were susceptible to ionomycin-induced apoptosis, as measured by viability. In contrast, BL2 cells latently infected with recombinant E2KO virus were protected from ionomycin-induced apoptosis (Figure 1B). These results confirm that the limited pattern of EBV gene expression associated with P3HR1, Oku-BL, Sal-BL and an E2KO virus is sufficient to enhance survival of ionomycin treated cells, that BIM is not involved in this pathway to apoptosis and that the p53 context is unimportant. Throughout, in order to confirm that death was a result of cells undergoing apoptosis, protein extracts prepared from treated and untreated cells were separated by SDS-PAGE and analysed by western blotting using an antibody which detects both full length poly(ADP-ribose) polymerase (PARP) and its cleaved fragments. PARP is proteolytically cleaved from a full-length 113 kDa protein to 89 kDa and 24 kDa fragments by caspases activated during apoptosis. PARP cleavage is widely accepted to be a hallmark of programmed cell death [29]. All these data are shown in supplementary Figure S1.

Having confirmed that the EBV-mediated protection of BL cells from ionomycin-induced apoptosis only requires the expression of a limited set of viral genes, the number of candidate genes was reduced further by assessing the response of cells that exhibit type I latency. This is a restricted form of EBV latency in which only EBNA1, the EBERs and BARTs are expressed, and is the EBV gene expression pattern found in the majority of primary EBV-positive BL. The Latency I BL-derived line, Mutu I, was used for investigation. Mutu cells that had drifted in culture to produce the full latency III pattern of EBV gene expression (Mutu III) were analysed for comparison. Figures 1C and S1 show that Mutu III cells were resistant to ionomycin-induced apoptosis and continued to proliferate. In contrast, fewer than 20% of Mutu I cells survived after 48 hours of exposure to ionomycin and complete cleavage of full-length PARP was observed, consistent with death by apoptosis in response to ionomycin. These data demonstrate that type I latency (EBNA1 and the EBERs) is insufficient to protect against ionomycin-induced apoptosis.

Since the experiments described so far utilized BL-derived cells, we determined whether two lymphoblastoid cell lines, LCL-BF and LCL-CH, established by infection of primary B cells with the B95-8 strain of EBV [30] show the same resistance to ionomycin as type III BL cells. Viable cell counts for both these LCLs showed that at least 90% of cells survived after 48 hours of exposure to ionomycin (Figure 1D). PARP is also unaltered by treatment in both LCLs, confirming that these LCLs failed to undergo apoptosis even after exposure to ionomycin for 48 hours (Figure S1). These results are in marked contrast to the response of LCLs to genotoxic drugs such as cisplatin and nocodazole, since EBV cannot protect against genotoxin-induced, p53-mediated apoptosis in LCLs [23].

### EBNA3A, EBNA3B and EBNA3C are not involved in protection against ionomycin-induced apoptosis

**Figure 2:**
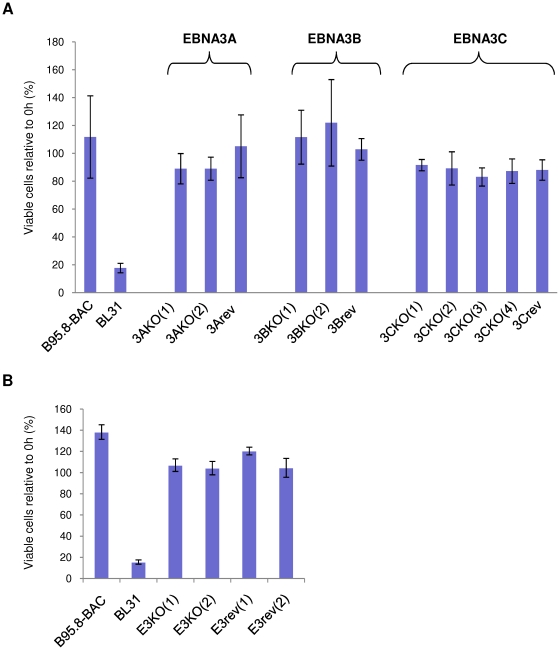
Deleting *EBNA3A*, *3B* or *3C* or the complete *EBNA3* locus has little or no effect on ionomycin-induced apoptosis. (**A**) EBV-negative BL31 cells and their converts infected with recombinant B95.8 EBV (WT), *EBNA3A, -3B* and -*3C*-knockout EBVs, or their respective revertants were treated with 1 µg/ml ionomycin (IM) for 48 hours. (**B**) EBV-negative BL31 cells and their converts infected with recombinant B95.8 EBV (WT), *EBNA3* locus knockout (E3KO) or revertant (E3rev) EBVs were treated with 1 µg/ml ionomycin for 48 hours. In both (A) and (B) viable cells that exclude trypan blue were counted after 48 hours and expressed as a percentage relative to the starting population. The mean and standard deviation from three independent experiments are shown.

Expression of both EBNA3A and EBNA3C is necessary to inhibit genotoxin-induced apoptosis in a p53-mutant background [21], [22], we therefore asked whether expression of EBNA3A, EBNA3B and EBNA3C is necessary for resistance to ionomycin in the context of latent virus infection. The EBV-negative parental BL31 cells, along with BL31 cells infected with BAC-derived EBNA3A, EBNA3B or EBNA3C knockout viruses (3AKO, 3BKO and 3CKO respectively), their revertants (3Arev, 3Brev and 3Crev respectively) or wild type (WT, derived from the prototype B95.8 strain) viruses (described in [22]) were treated with ionomycin. After 48 hours, more than 80% of the parental EBV-negative BL31 cells were dead (Figure 2A) and exhibited considerable cleavage of PARP (Figure S2). As expected, infection of BL31 cells with WT EBV or any of the revertant viruses improved survival dramatically. Interestingly – as predicted by Kelly *et al* [25] – BL31 cells infected with the 3AKO, 3BKO or 3CKO viruses were also resistant to ionomycin-induced apoptosis, with at least 90% of cells remaining viable after 48 hours. This survival was also reflected by absence of PARP cleavage in these cell lines (Figure S2). This demonstrates that in BL31 cells the absence of EBNA3A, EBNA3B or EBNA3C individually has little or no effect on the EBV-mediated protection against ionomycin-induced apoptosis and is in marked contrast to the contribution both EBNA3A and EBNA3C make to the EBV-mediated protection of similar BL cells against apoptosis induced by genotoxic drugs such as cisplatin [22]. In order to show that these observations were not specific to BL31 cells, experiments were repeated using a panel of BL2 cells (which lack BIM, but carry WT p53) infected with recombinant 3AKO, 3BKO, 3CKO, revertant or WT EBV. The results obtained from the BL2 panel of cells are similar to those obtained with the BL31 panel (data not shown). Together these results indicate that individually deleting EBNAs 3A, 3B or 3C does not alter the protection conferred by EBV against ionomycin-induced apoptosis. The data also confirm the observations that the apoptotic pathway induced by ionomycin is independent of both p53 and BIM.

While removing EBNA3A, EBNA3B or EBNA3C individually did not alter the capacity of EBV to protect against ionomycin, the possibility of functional redundancy between the EBNA3 proteins could not be ruled out. Therefore, a recombinant EBV harbouring a deletion of the entire EBNA3 locus (E3KO) was constructed and BL31 and BL2 cells were established by infection with E3KO or its revertant (E3rev) [31]. While few of the parental BL31 cells remain viable after 48 hours of exposure to ionomycin, BL31 cells infected with the E3KO, WT and revertant viruses are all resistant to ionomycin-induced apoptosis, as measured by viability and PARP cleavage (Figures 2B and S2). Experiments with a BL2 panel of cells produced essentially similar results (data not shown). All these results eliminate the possibility that the EBNA3s can functionally substitute for each other in the individual deletion viruses, and confirm that the EBNA3s are not involved in the EBV-mediated protection of BL cells from ionomycin-induced apoptosis.

### The BHRF1 locus provides partial resistance to ionomycin-induced apoptosis

**Figure 3:**
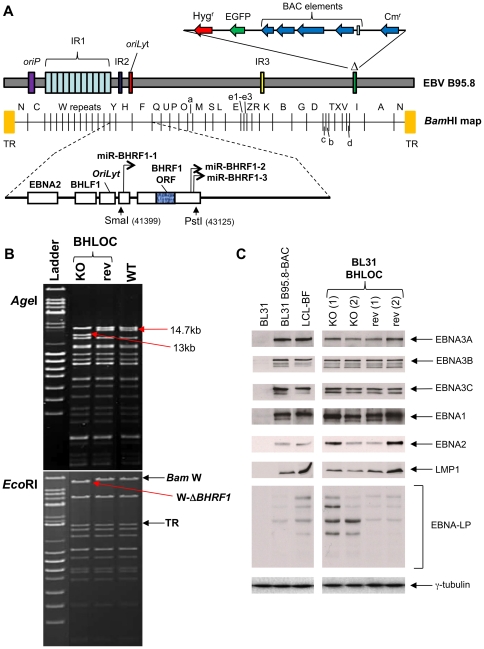
Construction of an EBV-BAC recombinant virus from which *BHRF1* locus has been deleted. (**A**) Schematic representation of the recombinant EBVs generated with a deletion in the *BHRF1* locus (*BHLOC* KO). Recombinant EBV-BACs harbouring a deletion of the complete *BHRF1* locus were constructed. The BAC region is inserted at the site of the 10 kb deletion of B95-8 (Δ). Vertical arrows indicate the restriction enzyme sites and positions (within the EBV genome sequence, Accession No V01555) between which the *BHRF1* locus was deleted. This region was reinserted to generate the revertant virus (*BHLOC* rev). The schematic is not drawn precisely to scale. (**B**) Validating BHRF1 knockout and revertant EBV-BACs. DNA from the *BHRF1* locus (BHLOC)-knockout (KO – BAC JY28+2.4) and its revertant (rev – BAC JY32+2.3) EBV-BACs as well as the wild-type EBV-BAC (WT) were analysed by restriction digestion with *Age*I (top) or *Eco*RI (bottom) followed by pulsed field gel electrophoresis. A mixture of λ-DNA *Bst*EII and λ-DNA mono cut mix ladders (NEB) were used as a size marker (Ladder). Bands whose sizes are changed by the deletion of the BHRF1 locus (13 kb AgeI fragment, and the *Bam* W-containing EcoRI fragment lacking *BHRF1* - W-Δ*BHRF1*) are indicated. The *Eco*RI restriction fragments that include the *Bam* W repeats and the terminal repeats (TR) are also indicated. (**C**). Protein extracts from BL31, BL31 *BHLOC* KO, revertants and an LCL were separated by SDS-PAGE and western immunoblotted with antibodies specific to the EBV proteins indicated (and the cellular protein γ-tubulin as a loading control).

Previous studies showed that the EBV viral BCL-2 homologue, BHRF1, is only expressed early upon infection of resting B cells or during lytic reactivation [16], [32]. Although ionomycin treatment does not consistently induce the viral lytic cycle in any of the panel of BL cells studied here (for examples see Figure S3), Kelly and colleagues have shown that BHRF1 can be expressed at a low level during latency in ‘Wp-restricted’ BL-derived cell lines (eg. Sal-BL and Oku-BL) and can suppress the apoptotic pathway activated by ionomycin in BL cells when it is delivered by transfection [25]. By western blotting with an anti-BHRF1 monoclonal antibody, we confirmed expression of BHRF1 in Oku-BL and Sal-BL, but detected no expression in various untreated or treated BL2, BL31 and BL41 converts or in Mutu III cells (Figure S3). However, one could not formally exclude the possibility that BHRF1 might be functionally active at a level that is undetectable by western blotting extracts from these latently infected cells. Therefore, using recombination in the B95-8 EBV-BAC, viruses were constructed from which the *BHRF1* locus was deleted (BHLOC KO) and subsequently replaced to produce a revertant virus (BHLOC rev – Figure 3A and B). The deletion removes not only the BHRF1 coding sequence, but also three miRNAs that have been shown to inhibit apoptosis early after infection of primary B cells [33]. The recombinant viruses were then used to infect EBV-negative BL31 cells and stably infected cells were selected using hygromycin and allowed to grow out as a bulk population. Attempts to produce BL2 lines infected with the BHLOC KO virus were unsuccessful for reasons we don't understand. Protein extracts from the BL31 cells established with these recombinant EBVs were analysed by western blotting using antibodies for each of the EBV latent proteins. It was confirmed that these cells display no consistent changes to the levels of the EBV latent proteins – EBNA1, EBNA2, EBNA3A, EBNA3B, EBNA3C and LMP1; however in the two BHLOC KO lines the amount of EBNA-LP appeared slightly elevated relative to wild type and revertant infections (Figure 3C) – this was also reported in a recent study focusing on the *BHRF1* miRNAs [34].

**Figure 4:**
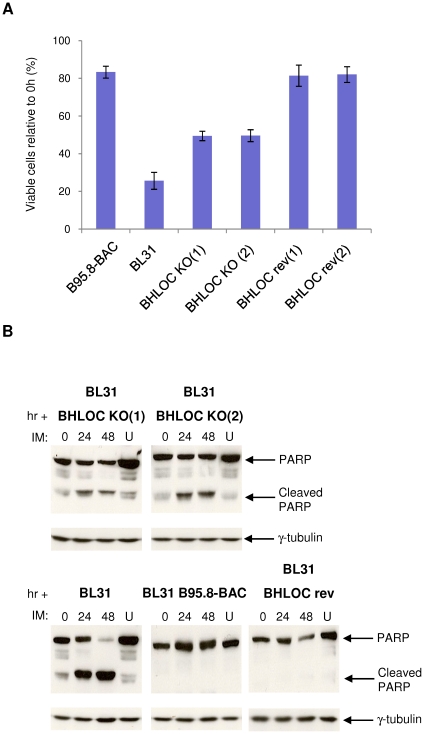
BL31 cells established using *BHLOC* KO become partially sensitive to ionomycin treatment. (**A**) EBV-negative BL31 and its EBV-positive converts established using recombinant B95.8 (WT), *BHRF1* locus-knockout (*BHLOC* KO) and *BHRF1* locus-revertant (*BHLOC* rev) EBVs were treated with 1 µg/ml ionomycin (IM) or DMSO (vehicle control). After 24 hours cell viability was determined using the CellTiter-Glo assay and expressed relative to vehicle-treated cells. The mean and standard deviation from three independent experiments are shown. (**B**) Protein extracts from cells harvested at 24-hour intervals were separated by SDS-PAGE and analysed by western immunoblotting using antibodies that detect full-length and cleaved PARP. Ionomycin-treated cells are compared to the same cells treated with vehicle control for 48 hours (U) and γ-tubulin was used as a loading control.

In order to determine the contribution made by the *BHRF1* locus to EBV-mediated protection against ionomycin-induced apoptosis, the response to ionomycin of BL31 carrying BHLOC KO or BHLOC rev viruses were examined. Figure 4A shows that 24 hours after exposure to ionomycin, only 25% of EBV-negative BL31 cells remain viable, compared to >80% of BL31 cells infected with the WT-EBV or BHLOC rev. It was apparent from multiple experiments that BL31 cells infected with the BHLOC KO virus showed partial resistance to ionomycin-induced apoptosis – with about 50% of the cell population remaining viable after 24 hours. Western blotting for PARP echoes this result, with a substantial amount of cleaved PARP detectable in EBV-negative BL31 cells, very little or no cleaved PARP in WT- or BHLOC rev-infected BL31 cells, and an intermediate level of PARP cleavage in BL31-BHLOC KO (Figure 4B). These data indicate that the *BHRF1* locus is involved in the EBV-mediated protection of BL cells against ionomycin-induced apoptosis. However, since the loss of the *BHRF1* locus consistently resulted in only a partial loss of protection, it is likely that another EBV gene product(s) is also involved in the resistance to ionomycin.

### EBV inhibits the induction of NOXA by ionomycin

**Figure 5:**
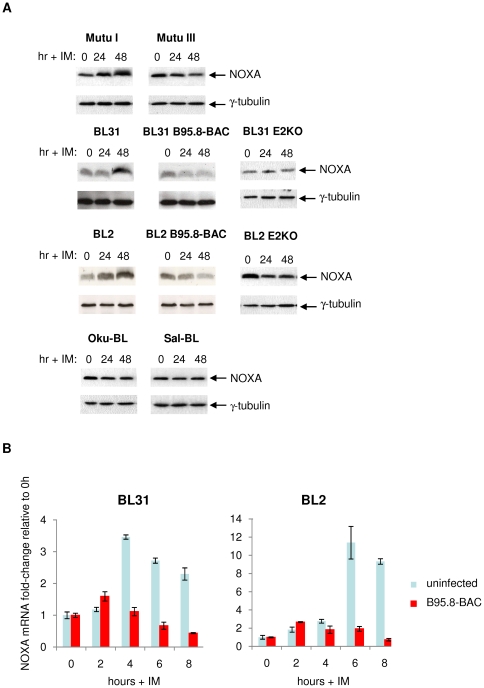
Ionomycin induces accumulation of NOXA protein and mRNA in EBV-negative and latency I BL cells, but not in EBV-positive cells that are resistant to ionomycin-induced apoptosis. (**A**) Protein extracts from the cells indicated before and after exposure to 1 µg/ml ionomycin (IM) for up to 48 hours were separated by SDS-PAGE and analysed by western immunoblotting using antibodies directed against NOXA. Throughout, γ-tubulin was used as a loading control. (**B**) EBV inhibits the accumulation of NOXA mRNA induced by ionomycin. EBV-negative and recombinant B95.8 (WT) EBV-converted BL31 and BL2 cells were exposed to 1 µg/ml ionomycin for up to 8 hours. Cells were harvested at 2-hour intervals and RNA was extracted. The extracts were analysed by quantitative RT-PCR and NOXA mRNA expression was normalized to that of UBC mRNA, which is stably expressed during ionomycin treatment/apoptosis over the time period used.

To identify cellular factors that might be involved in the apoptotic pathway triggered by ionomycin in BL cells and blocked by EBV, we surveyed the changes in expression of various pro-apoptotic proteins in BL cells exposed to ionomycin. Initially BAD, BID, PUMA and NOXA were investigated by western blotting (Figures 5A, S3 and S4). Although the steady-state level of NOXA varied little between the various cell lines used here, it was the only one of these pro-apoptotic factors that consistently showed an increase in response to ionomycin (Figures 5A and S4). The gene encoding NOXA is a known transcriptional target of the tumour suppressor p53 and its expression is upregulated during p53-mediated cell death [35]. However, western blot analysis reproducibly showed an increase in NOXA protein levels in Mutu I and EBV-negative BL31 cells exposed to ionomycin and since both lines express only mutant p53 [22], [36], this indicates that the induction is not dependent on p53-mediated transactivation. In contrast, NOXA protein levels were unaffected (or decreased) in Mutu III cells, BL31 cells latently infected with wild type B95.8 recombinant virus or infected with the P3HR1-like, EBNA2 knockout virus (E2KO). This indicates that only a limited pattern of EBV gene expression is necessary to block NOXA accumulation. In a similar BL2 series of cells and Oku- and Sal-BL cells it was only in the EBV-negative BL2 cells that NOXA accumulated and apoptosis was induced in response to ionomycin. These cells all carry wild type, transactivation competent p53 [22], but ionomycin did not induce its stabilization (data not shown), again consistent with NOXA induction being p53-independent. Quantitative reverse transcriptase PCR (qRT-PCR) analysis of RNA from BL31 and BL2 cells established that this induction of NOXA expression involves the modulation of NOXA mRNA and may therefore be regulated at the level of transcription; in both the EBV-infected converts this induction is inhibited (Figure 5B). Together these data indicate that NOXA is consistently up-regulated in EBV-negative and latency I BL-derived cells that undergo apoptosis in response to ionomycin. The accumulation of NOXA is independent of p53-status and can be inhibited by a factor(s) encoded by EBV in latency state III, but does not seem to require EBNA2, (or the LMPs) that are absent from the E2KO-infected cells, Oku- BL and Sal-BL.

### NOXA contributes to ionomycin-induced apoptosis

**Figure 6:**
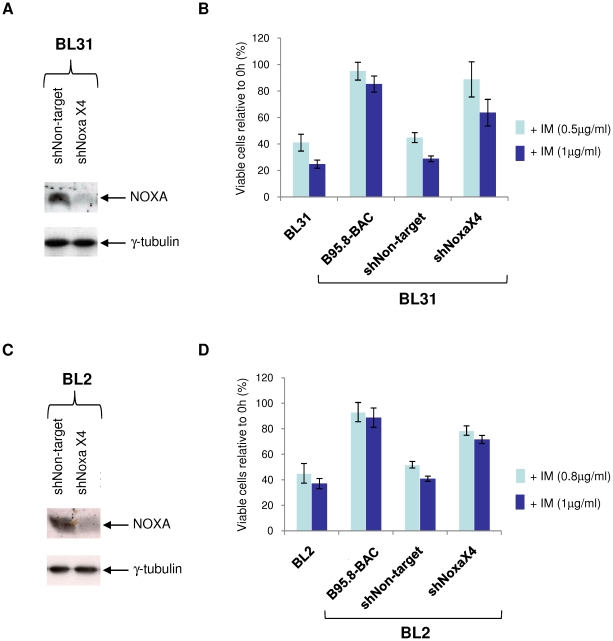
shRNA-mediated knockdown of NOXA protects against ionomycin-mediated apoptosis. BL31 cells were transduced with multiple (shNOXAx4) lentiviral vectors carrying shRNA directed against NOXA mRNA, or with a non-target shRNA control (shNon-target) lentivirus. (**A**) Western immunolotting shows the reduction in basal NOXA expression due to the shRNAs; γ-tubulin was used as a loading control. (**B**) BL31-shNOXAx4 and BL31-shNon-target, EBV-negative BL31 and its B95.8 (WT) EBV convert were exposed to DMSO (vehicle control), or 0.5 or 1 µg/ml ionomycin (IM) for 24 hours. Cell viability was determined by CellTiter-Glo assay and expressed as a percentage of vehicle-treated controls. The mean and standard deviation of three independent experiments is shown. (**C**) BL2 cell lines transduced with lentiviruses as (A) above, and immunoblotted to show levels of NOXA depletion. (**D**) BL2 cell lines treated with ionomycin and assayed as described in (B).

NOXA and its mRNA are consistently up-regulated in EBV-negative BL cells that undergo apoptosis in response to ionomycin. However, NOXA expression appears to be largely unaffected in EBV-positive BL cells that are resistant to ionomycin. It was therefore important to determine whether NOXA is necessary for ionomycin-induced apoptosis in sensitive BL cells. To this end, four lentiviral vectors expressing shRNAs targeted against NOXA mRNA were used to transduce EBV-negative BL31 and BL2 cells. None of the lentiviruses alone achieved greater than a 40–50% reduction in protein level (data not shown). However, transduction of BL31 and BL2 cells with all four shNOXA lentiviruses (shNOXAx4) achieved a substantial reduction in NOXA expression in both cell lines. BL31 and BL2 cells were also transduced with the non-targeting shRNA-expressing lentiviruses to establish lines as negative controls (Figure 6A & C).

BL31- and BL2-shNOXAx4 and shNon-target cell lines were then exposed to ionomycin and their responses compared to those of EBV-negative and WT EBV-converted BL31 and BL2. In both BL31 and BL2 cell lines, cell viability after ionomycin treatment is substantially improved as a result of NOXA knock-down, although not quite restored to the protection offered by EBV infection (Figures 6B and D). Similarly, PARP cleavage is reduced or prevented in the absence of NOXA, in both BL31 and in BL2 cells (Figure S5). These observations are consistent with either an incomplete ‘knockdown’ of NOXA, or with the possibility that NOXA represents a partial component of the apoptotic stimulus caused by ionomycin, but either way, confirm that NOXA makes a major contribution to the apoptosis induced in these cells by ionomycin.

### Neither the EBNA3s nor the *BHRF1* locus contribute to the inhibition of NOXA induction

**Figure 7:**
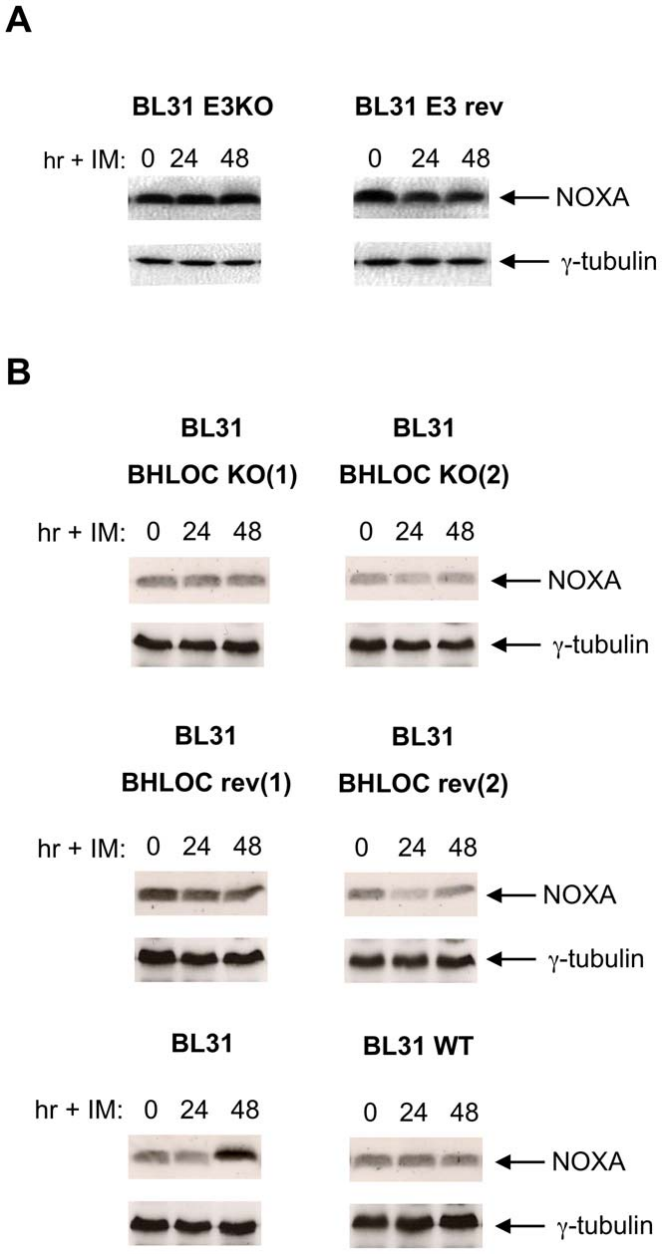
Neither the *EBNA3* nor the *BHRF1* loci are necessary to block the induction of NOXA. (**A**) A comparison of NOXA induction in *EBNA3KO* and *EBNA3* revertant BL31 cells after treatment with ionomycin (IM) and analysis by western immunoblotting. (**B**) Similar analysis of *BHLOC* KO BL31 lines and a revertant line. Induction of NOXA by ionomycin in BL31 is shown for comparison. Throughout γ-tubulin was used as a loading control.

Since BL31 cells latently infected with an EBNA3 knockout virus (E3KO) are resistant to ionomycin, it was predictable that EBV carrying this deletion would still block NOXA accumulation. This is formally shown in Figure 7A. However, more surprising was the observation that virus with the *BHRF1* locus deletion – despite providing reduced resistance to ionomycin treatment – also suppressed NOXA accumulation as effectively as revertant and WT B95.8 EBVs (Figure 7B).

### Responses of BL-derived cells to staurosporine and etoposide confirm two distinct pathways

Staurosporine is a broad-spectrum protein kinase inhibitor, is widely used as an inducer of apoptosis in a variety of cell types, but is chemically unrelated to ionomycin [37]. Etoposide is a DNA topoisomerase II inhibitor that can induce apoptosis through DNA damage-induced p53 activation [38].

**Figure 8:**
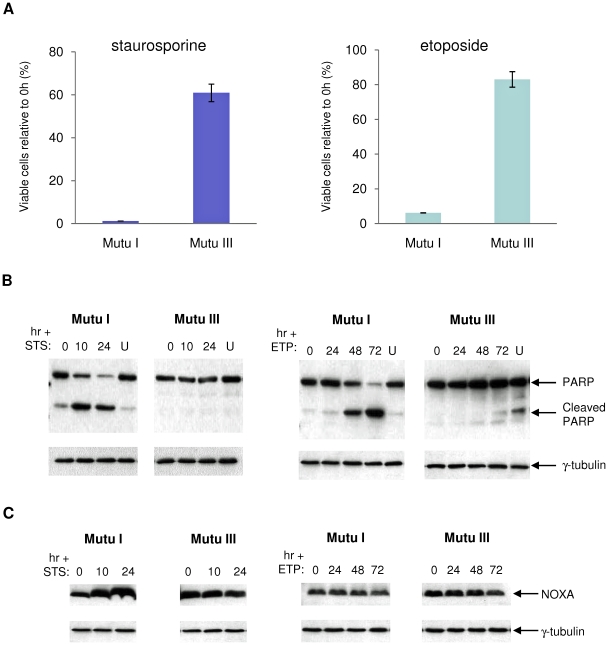
Comparison of staurosporine- and etoposide-induced apoptosis in Mutu BL cells. Mutu I and Mutu III cells were exposed to 0.25 µM staurosporine (STS) or 800 ng/ml etoposide (ETP) for 24 hours or 72 hours respectively. (**A**) Cell viability was determined using the CellTiter-Glo assay and expressed as a percentage of DMSO vehicle-treated cells. The mean and standard deviation of three independent experiments is shown. Protein was extracted from cells harvested at the start of treatment and the times indicated. Western immunoblotting was performed using antibodies that detect PARP (**B**) or NOXA (**C**). U indicates vehicle-treated control cells after 24 or 72 hours, and γ-tubulin was used as a loading control.

**Figure 9:**
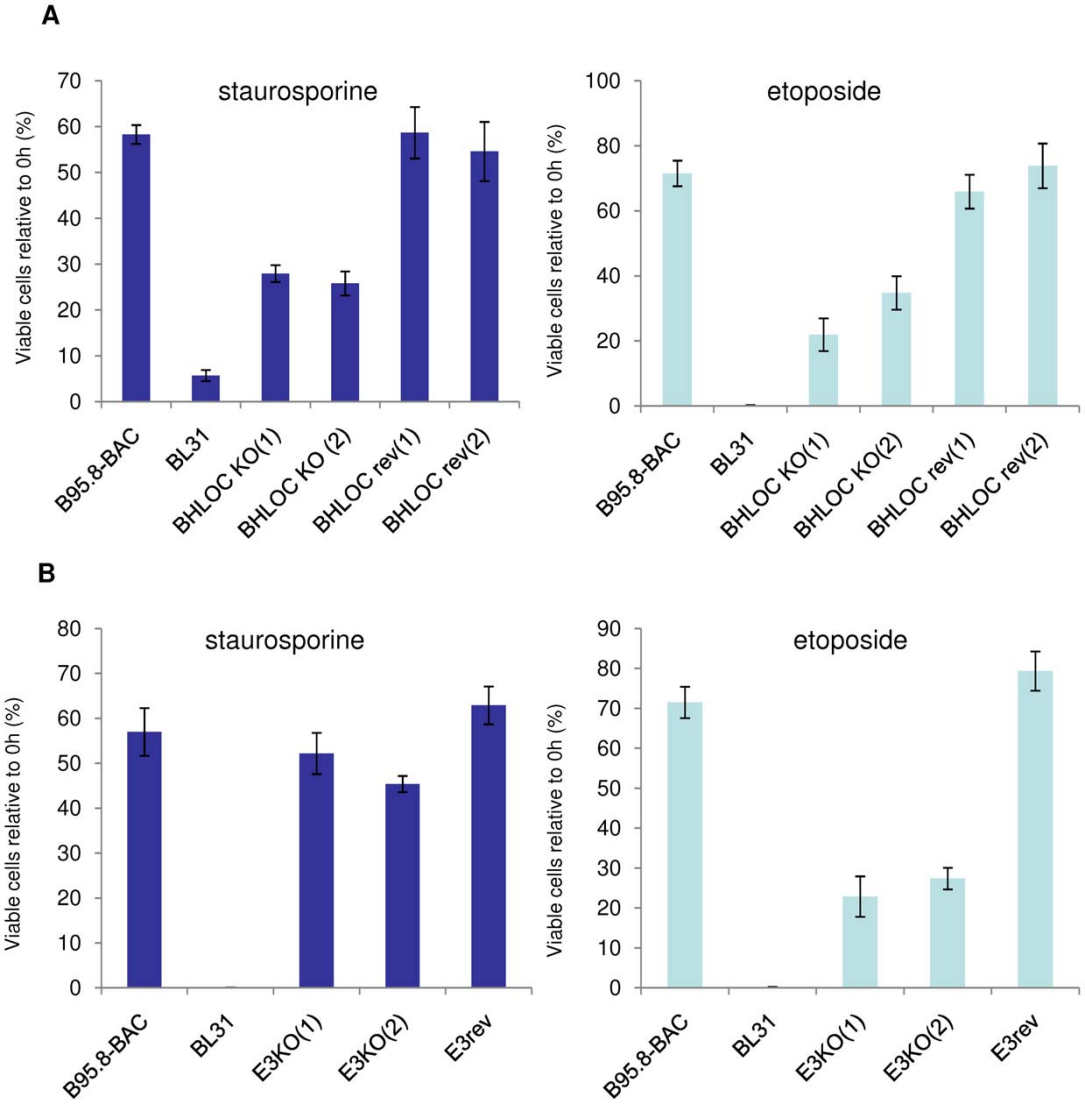
The *BHRF1* locus makes a significant contribution to resistance against both staurosporine and etoposide, but the EBNA3s only protect against genotoxin-induced damage. (**A**) EBV-negative BL31, its recombinant B95.8 (WT) EBV convert and BL31 cell lines established with recombinant *BHRF1* locus-knockout (*BHLOC* KO) and revertant (*BHLOC* rev) EBVs were treated with 0.25 µM staurosporine (STS, left panel) or 800 ng/ml etoposide (ETP, right panel) for up to 24 hours. Cell viability was determined after 24 hours using the CellTiter-Glo assay and expressed as a percentage of DMSO vehicle-treated cells. (**B**) Experiments and analysis described in (A) performed on BL31 converts made with EBNA3 locus knockouts (E3KO) and revertant virus.

Similar experiments to those described above were performed to assess the responses of BL-derived cells to staurosporine and etoposide in order to establish whether they could be categorised with either ionomycin or the genotoxins. Initially Mutu I and Mutu III were compared. Although both staurosporine and etoposide induced apoptosis in Mutu I, latency-III EBV gene expression (in Mutu III) substantially enhanced survival after both treatments (Figure 8A & B). Staurosporine induced NOXA in sensitive Mutu I cells, but in contrast, etoposide had no effect on NOXA levels and must therefore activate a separate pathway (Figure 8C). When the BHLOCKO infected cells were tested, they exhibited partial resistance to both staurosporine and etoposide, but as expected, the BHLOC KO virus still blocked NOXA accumulation (Figures 9A and S6). It seems that the *BHRF1* locus enhances survival irrespective of the type of trigger, but acts independently of NOXA.

When BL31 cells infected with the *EBNA3* locus knockout (E3KO) series were analysed in similar assays, the deletion viruses showed similar levels of protection to B95.8-BAC (WT) EBV in response to staurosporine. In contrast when the cells were treated with etoposide, only 10–20% of the E3KO-infected cells survived, but NOXA was not induced (Figure 9B and S7). These observations suggest that staurosporine induces apoptosis in BL cells by activating the same or a similar pathway to that activated by ionomycin whereas etoposide works, like cisplatin, via a second distinct pathway associated with DNA damage.

## Discussion

Previous work in our laboratory showed that the resistance conferred by EBV against cell death induced by agents that cause damage to DNA, including the mitotic spindle poison nocodazole and the DNA cross-linking agent cisplatin, is dependent on the co-expression of EBNA3A and EBNA3C [22]. Here we have expanded this group to include etoposide (see below). Protection correlates with the down-regulation of the BH3-only pro-apoptotic protein BIM by the combined action of EBNA3A and EBNA3C, but is only apparent in the absence of a functional p53. When BIM is not expressed, but p53 is functional (as for instance in BL2 cells), latent EBV does not appear to provide any survival advantage against apoptosis induced by these genotoxic drugs. These findings appeared to be inconsistent with the observations made by Kelly and colleagues that EBV protects both BL2 cells (that carry WT p53, but are BIM-negative) and BL31 cells (that carry mutant p53, but are BIM-positive) to a similar extent when apoptosis was induced by the calcium ionophore, ionomycin [20], [25]. We hypothesised that ionomycin must activate a distinct apoptotic pathway in BL cells that is both independent of p53 status and not associated with BIM. We confirm here that latent EBV protects cells from ionomycin and also the apparently unrelated drug staurosporine via a similar pathway and show that EBV blocks the accumulation of the pro-apoptotic protein NOXA that is induced by both of these agents.

The data from BL41 cells infected with P3HR1 virus and BL2 cells infected with a recombinant virus that also fail to express EBNA2 (E2KO) were consistent with a restricted pattern of viral gene expression – EBNA1, EBNA3A, EBNA3B, EBNA3C, truncated EBNA-LP, the EBER and the BART RNAs that remain in B95.8 being sufficient for protection from ionomycin. We obtained similar results with Oku-BL and Sal-BL that carry a virus with a similar genomic deletion to P3HR1. If EBV gene expression was restricted further, as in Mutu I cells (EBNA1, EBERs and BARTs only) there was no protection. Analyses utilizing recombinant EBVs carrying specific gene deletions of EBNA3A, EBNA3B, EBNA3C or the whole *EBNA3* locus showed that none of these genes is necessary for the survival phenotype and that there is no functional redundancy – that is, one EBNA3 cannot substitute for either of the others.

It was recently shown that BHRF1 might not be a strictly ‘lytic’ product and that the genomic locus from which it derives is also the source of three potentially anti-apoptotic miRNAs [16], [25], [33]. We therefore constructed a recombinant EBV from which the complete *BHRF1* locus was deleted to formally test whether these potentially anti-apoptotic factors are required for protection of latently infected cells against ionomycin (and staurosporine). Deletion of the *BHRF1* locus partially sensitized latently infected BL31 cells to these drug treatments, indicating that the locus must be involved in mediating resistance. However at this stage we cannot say whether this is due to the action of BHRF1 protein (a BCL-2 homologue) or the *BHRF1* miRNAs or both. Furthermore, since the reduction in survival is only partial, this implies that another EBV factor(s) contributes to the resistance phenotype by an as yet unidentified mechanism.

BH3-only proteins are critical mediators of the intrinsic apoptotic pathway (reviewed in [39]). Therefore, in order to identify cellular factors that might be associated with the apoptotic pathway activated by ionomycin (and staurosporine), the expression of pro-apoptotic BH3-only members of the BCL-2 family – BAD, BID, NOXA and PUMA – was investigated. Of these NOXA was the only protein consistently induced in EBV-negative and latency I BL cells that were sensitive to treatment with both ionomycin and staurosporine. In contrast, up-regulation of NOXA did not occur in any EBV-positive BL cells exhibiting resistance to the drugs. These data suggested that apoptosis triggered by ionomycin and staurosporine involves the induction of NOXA expression and that EBV rescues BL cells from apoptosis, at least in part, by inhibiting the accumulation of NOXA. ShRNA-mediated knock-down of NOXA expression was able to substantially enhance the survival of EBV-negative BL31 and BL2 cells challenged with ionomycin, confirming that ionomycin-induced apoptosis is at least partly mediated by NOXA and that inhibiting its expression can promote B cell survival. The presence of shRNA against NOXA did not prevent its induction by ionomycin, but since NOXA was induced from a lower baseline level, the improved survival manifested as delayed apoptosis (apparent at 24 hours post treatment) rather than as complete resistance – if the cells were left long enough they died. We assume the rate at which new RNA was synthesised outstripped the capacity of the shRNA to inactivate NOXA message. Despite these technical drawbacks inherent in shRNA experiments, the results were highly reproducible and consistent in two different cell backgrounds.

It was more surprising to see that NOXA levels did not increase in EBV-positive BL cells lacking the *BHRF1* locus, since they are partially sensitive to ionomycin and staurosporine treatment. This suggests that these drugs trigger an additional pro-apoptotic mechanism in addition to NOXA induction, which is blocked by the active component of the *BHRF1* locus – this could be BHRF1, expressed at a low level as hypothesised by Kelly *et al*, mimicking BCL-2 and neutralizing multiple pro-apoptotic factors.

The increase in NOXA protein after drug-treatment was mirrored by an increase in NOXA mRNA, suggesting that the regulation is at the level of RNA turnover or transcription. Since wild type p53 is absent from BL41, BL31 and Mutu I cells the induction of NOXA expression by ionomycin does not involve p53-mediated activation of *NOXA* transcription. Consistent with this, ionomycin does not induce the stabilization and accumulation of wild type p53 in BL2 cells (data not shown). However, it is currently unclear by what signalling pathway ionomycin (or staurosporine) induces the accumulation of NOXA. Furthermore, using the various mutants of EBV described above, we were unable to identify the viral gene or gene product(s) that is responsible for the block in NOXA accumulation. None of the latency-associated proteins, the EBERs, BHRF1, the *BHRF1* miRNAs or most of the BART miRNAs, appear to be necessary to block the ionomycin-induced increase in NOXA. Of the currently recognised latency-associated gene products only the BART miRNAs that are not missing from the B95.8 genome (miR-BARTs 1, 2, 3, 4 and 15 [40], or the W1W2 repeat region of EBNA-LP [15] remain as candidates for this regulation. However, at this stage we cannot formally exclude a contribution by products from other regions of the EBV genome not presently associated with latency.

Finally, etoposide – a DNA topoisomerase II inhibitor and therefore another drug that can cause damage to DNA – also triggered apoptosis that was blocked by latency III EBV gene expression in Mutu and BL31 cells. Apoptosis did not involve the induction of NOXA, but resistance required expression of EBNA3A and EBNA3C, in this respect etoposide has a similar mode of action to cisplatin and nocodazole. These data are consistent with the hypothesis of two distinct pathways and suggest that EBNAs 3A and 3C might play a specific role in the DNA damage response, in addition to the regulation of apoptosis *per se*.

## Materials and Methods

### Cell culture

Burkitt's lymphoma-derived cell lines: BL41, BL41/B95.8, BL41/P3HR1, Oku-BL, Sal-BL, BL2, BL31, Mutu I-clone 179 and Mutu III [described in [21], [22]; lymphoblastoid cell lines: LCL-BF and LCL-CH (described in [24], [30]) and HEK293 cells [31] were all cultured in RPMI 1640 medium containing L-glutamine (Invitrogen, UK) supplemented with 10% fetal calf serum and penicillin-streptomycin at 37°C in a humidified incubator with 10% CO_2_. BL2 and BL31 cell lines were additionally supplemented with 1 mM sodium pyruvate (Sigma, UK) and 50 mM α-thioglycerol (Sigma). Hygromycin B (Roche, UK) was added at a concentration of 100 µg/ml to all BL cell lines containing recombinant hygromycin-resistant EBV. BL2 cells infected with a recombinant EBNA2-knockout virus (BL2+E2KO) [20]. For routine passage, cells were split 1∶3 every 2 or 3 days. For experiments, all cells were re-suspended in fresh medium at a density of 3×10^5^ cells/ml 24 hours prior to manipulation.

### Generation and use of recombinant viruses

BL31 and BL2 cell lines carrying recombinant EBNA3 mutants and their revertants were described previously [22], [31]. EBV containing a deletion of the *BHRF1* locus was generated using recA-mediated recombination. Briefly, the region of the EBV genome containing the *BHRF1* locus was sub-cloned, and digested with SmaI/PstI, treated with Klenow and re-ligated, to remove the fragment corresponding to positions 41399–43125 of the EBV genome (Accession number V01555). Approximately 500 bp on each flank of this deletion enabled homologous recombination with the B95-8 EBV-BAC genome [41], using a markerless insertion technique [42], to generate BHLOC KO EBV. Revertants of this (BHLOC rev) were generated using the same region of EBV lacking the deletion, which was reintroduced into the EBV-BAC genome to revert the mutant to the wild-type sequence. Mutant and revertant viruses were tested for the presence/absence of the desired mutation, and screened for undesired deletions/alterations by restriction digestion and pulsed-field gel electrophoresis. Recombinant EBV-BAC was transfected into HEK-293 cells that were selected with hygromycin, cloned, screened and used to generate virus as described elsewhere [43]. These recombinant viruses were used to infect BL31 and BL2 cells as described previously [22], [31].

### Induction of cell death and measurement of cell viability

Ionomycin (Sigma), staurosporine (Sigma) and etoposide (Calbiochem) were reconstituted in DMSO to form stock solutions. Ionomycin was added to the cell medium to give a final concentration of 1 µg/ml unless otherwise stated. Staurosporine was used at a final concentration of 0.25 µM and etoposide was used at a final concentration of either 500 or 800 ng/ml. Viable cell counts were performed using a hemocytometer and based on the exclusion of trypan blue dye, or in some experiments, cell viability was measured using the CellTiter-Glo™ Luminescent Cell Viability Assay (Promega) according to the manufacturer's instructions. In this assay ATP activity is used as a measure of viable cells.

### Western blot analysis

This was performed essentially as previously described [44]. Briefly, protein extracted using RIPA buffer were resolved by sodium dodecyl sulfate-polyacrylamide gel electrophoresis (SDS-PAGE) and transferred to Protran nitrocellulose membranes (Schleicher & Schuell Bioscience). Membranes were blocked with 5% skimmed-milk in phosphate buffered saline (PBS) containing 0.05% Tween 20, probed with appropriate primary and HRP-conjugated secondary or tertiary antibodies. An ECL kit (Amersham Biosciences) was used for visualization. Primary antibodies were used according to the manufacturer's instructions: rabbit polyclonal anti-poly(ADP-ribose) polymerase or PARP (Roche), mouse monoclonal anti-γ-tubulin (GTU-88; Sigma), mouse monoclonal anti-NOXA (114C307; Calbiochem), rabbit polyclonal anti-BIM (Stressgen), rabbit polyclonal anti-PUMA (Sigma), mouse monoclonal anti-BID (clone 40, BD Transduction Laboratories), rabbit polyclonal anti-BAD (Cell Signaling), mouse monoclonal anti-BHRF1 (5B11; Chemicon), sheep polyclonal anti-EBNA3A (Exalpha), sheep polyclonal anti-EBNA3B (Exalpha), mouse monoclonal anti-EBNA3C (A-10), mouse monoclonal anti-LMP1 (CS1–4; DAKO), mouse monoclonal anti-EBNA2 (PE2; DAKO), mouse monoclonal anti-EBNA-LP (JF186; [45]) and human serum that recognizes EBNA1 (a gift from Paul Farrell). Human serum EE was from a patient with chronic infectious mononucleosis and has a very high titre of antibodies recognizing EBV early lytic antigens [46]. The human sera were used at a concentration of 1/10,000.

### Lentiviral-mediated shRNA

Lentiviruses carrying shRNA directed against NOXA [MISSION® shRNA, Sigma (TRCN0000: 150555,151311,153637,155570)] were used to transduce EBV-negative BL31 and BL2 cells at an MOI of 10. A non-targeting shRNA lentiviral vector was used as a negative control. Transduced cells were selected in puromycin (1 µg/ml) and the amount of NOXA depletion achieved by shRNAs was assessed by western blotting and real-time quantitative RT-PCR analyses (data not shown).

### Real-time quantitative RT-PCR

Total cellular RNA was extracted using RNeasy Mini kits (Qiagen) as per the manufacturer's instructions. Real-time quantitative RT-PCR was performed in triplicate on 0.1 µg RNA using the QuantiFast SYBR Green RT–PCR kit (Qiagen) and ABI 7900HT Fast Real-Time PCR system (Applied Biosystems). NOXA mRNA was quantified relative to ubiquitin C (UBC) mRNA using the following primer sets: NOXA (GAACGCGCCAGTGAACCCAA and CTTTGTCTCCAATCCTCCGG) and UBC (ATTTGGGTCGCGGTTCTTG and TGCCTTGACATTCTCGATGGT). The cycling conditions used were 95°C for 10 s followed by 60°C for 30 s.

## Supporting Information

Figure S1 **Latent EBV inhibits ionomycin-induced apoptosis in a variety of cell backgrounds.** BL41 and its EBV-positive counterparts, BL41/B95.8 and BL41/P3HR1, two Wp-restricted BL Oku-BL and Sal-BL, BL2 and its EBV-positive convert established using a recombinant *EBNA2*-knockout (E2KO) virus, latency I and latency III Mutu, and two lymphoblastoid cell lines (LCLs) were all exposed to 1 µg/ml ionomycin (IM) for up to 48 hours. Protein was extracted from cells harvested at the start of the treatment and the times indicated. Protein extracts were separated by SDS-PAGE and analysed by western blotting using antibodies which detect full-length and cleaved poly(ADP-ribose) polymerase or PARP. U indicates untreated negative control cells and C indicates treated cells (BL2 plus IM) after 48 hours used as a positive control. Throughout, γ-tubulin was used as a loading control.
(TIF)

Figure S2 **The** ***EBNA3*** **locus is not involved in the EBV-mediated protection against ionomycin-induced apoptosis.** The EBV-negative BL31 cell line, its recombinant B95.8-BAC EBV convert, BL31 cell lines established using individual *EBNA3A*-, *3B*- and *3C*-knockout viruses and their respective revertants, as well as BL31 cells lines converted with recombinant *EBNA3* locus-knockout (E3KO) and revertant (E3rev) EBVs were all treated with 1 µg/ml ionomycin (IM) for up to 48 hours. Protein was extracted from cells harvested at the start of the treatment and at the times indicated. Protein extracts were separated by SDS-PAGE and analysed by western blotting using antibodies that detect full-length and cleaved PARP. U indicates untreated control cells after 48 hours. Throughout, γ-tubulin was used as a loading control.
(TIF)

Figure S3 **Rescue from apoptosis induced by ionomycin is not dependent on EBV lytic gene expression and Wp-restricted BL lines express BHRF1.** Various EBV-positive BL cells were treated with 1 µg/ml ionomycin (IM) for 48 hours and harvested at the times indicated. Protein extracts from treated cells were separated by SDS-PAGE and analysed by western blotting using (**A**) the human serum EE which detects lytic antigens and (**B**) and (**C**) monoclonal antibodies against BHRF1 and BZLF1. Extracts from Akata cells [47] left untreated (**−**) or treated with anti-Ig [+ or (+)] to stimulate the expression of EBV lytic proteins were included to show lytic gene expression. BZLF1 was used as a control for lytic activation and γ-tubulin was used a loading control.
(TIF)

Figure S4 **Ionomycin does not consistently induce expression of BAD, BID or PUMA.** Protein extracts from representative BL-derived cells treated with 1 µg/ml ionomycin (IM) for up to 48 hours were analysed by western blotting using antibodies directed against the pro-apoptotic BH3-only factors PUMA, BAD and BID. Throughout, γ-tubulin was used as a loading control.
(TIF)

Figure S5 **shRNA-mediated knockdown of NOXA increases resistance to ionomycin-induced apoptosis.** BL31 and BL2 cells established using lentiviral vectors expressing shRNA targeted against NOXA were exposed to ionomycin (IM) at the concentrations indicated for up to 48 hours. BL cells established using lentiviruses expressing a non-targeting shRNA were included as controls. Protein was extracted from cells harvested at the start of the treatment and the times indicated. Western blotting was performed for evidence of PARP cleavage. U indicates vehicle-treated control cells after 48 hours. Throughout, γ-tubulin was used as a loading control.
(TIF)

Figure S6 **The** ***BHRF1*** **locus contributes to the EBV-mediated protection against apoptosis induced by both staurosporine and etoposide.** EBV-negative BL31, its recombinant B95.8-BAC EBV convert and BL31 cell lines established with recombinant *BHRF1* locus-knockout (BHLOC KO) and revertant (BHLOC rev) EBVs were treated with 0.25 µM staurosporine (STS) for up to 24 hours or 500 ng/ml etoposide (ETP) for up to 48 hours. Protein was extracted from cells harvested at the start of the treatment and the time points indicated. Western blotting was performed using antibodies directed against PARP (top and bottom panels) and directed against NOXA (middle panel). U indicates vehicle-treated control cells after 24 or 48 hours. Throughout, γ-tubulin was used as a loading control.
(TIF)

Figure S7 **The** ***EBNA3*** **locus protects against etoposide (genotoxin)-, but not staurosporine-induced apoptosis.** Similar experiments and analysis to (S6) were performed using BL31 cell lines established using recombinant *EBNA3* locus-knockout (E3KO) and revertant (E3rev) EBVs.
(TIF)
