# Supplementary figures and images for: Latent Epstein-Barr Virus Can Inhibit Apoptosis in B Cells by Blocking the Induction of NOXA Expression
Source: PLoS One. 2011 Dec 9;6(12):e28506. doi: 10.1371/journal.pone.0028506 (PMC3235132; doi:10.1371/journal.pone.0028506)

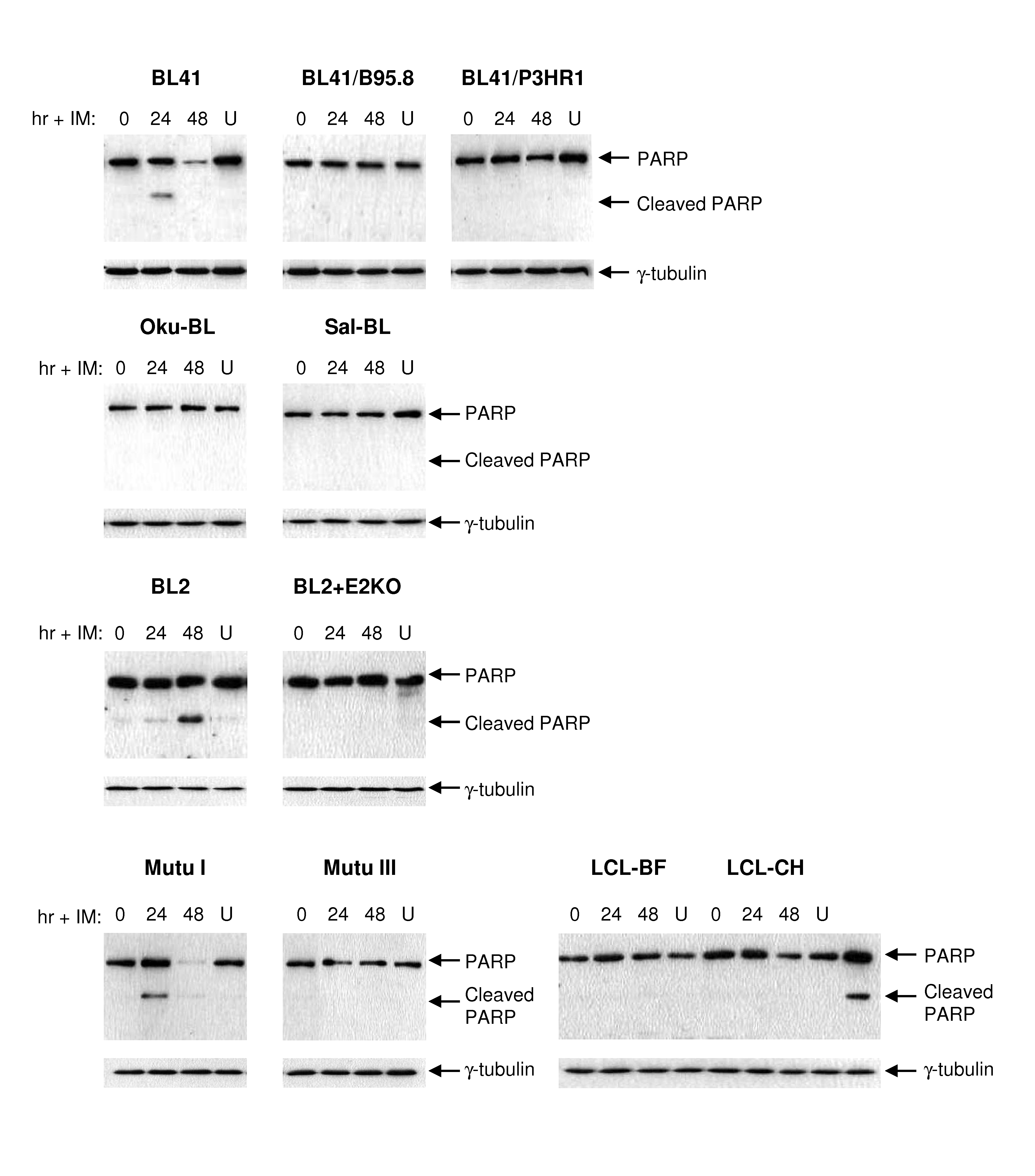

Supplement: Figure S1 — Latent EBV inhibits ionomycin-induced apoptosis in a variety of cell backgrounds. BL41 and its EBV-positive counterparts, BL41/B95.8 and BL41/P3HR1, two Wp-restricted BL Oku-BL and Sal-BL, BL2 and its EBV-positive convert established using a recombinant EBNA2-knockout (E2KO) virus, latency I and latency III Mutu, and two lymphoblastoid cell lines (LCLs) were all exposed to 1 µg/ml ionomycin (IM) for up to 48 hours. Protein was extracted from cells harvested at the start of the treatment and the times indicated. Protein extracts were separated by SDS-PAGE and analysed by western blotting using antibodies which detect full-length and cleaved poly(ADP-ribose) polymerase or PARP. U indicates untreated negative control cells and C indicates treated cells (BL2 plus IM) after 48 hours used as a positive control. Throughout, γ-tubulin was used as a loading control. (TIF) [file pone.0028506.s001.tif]

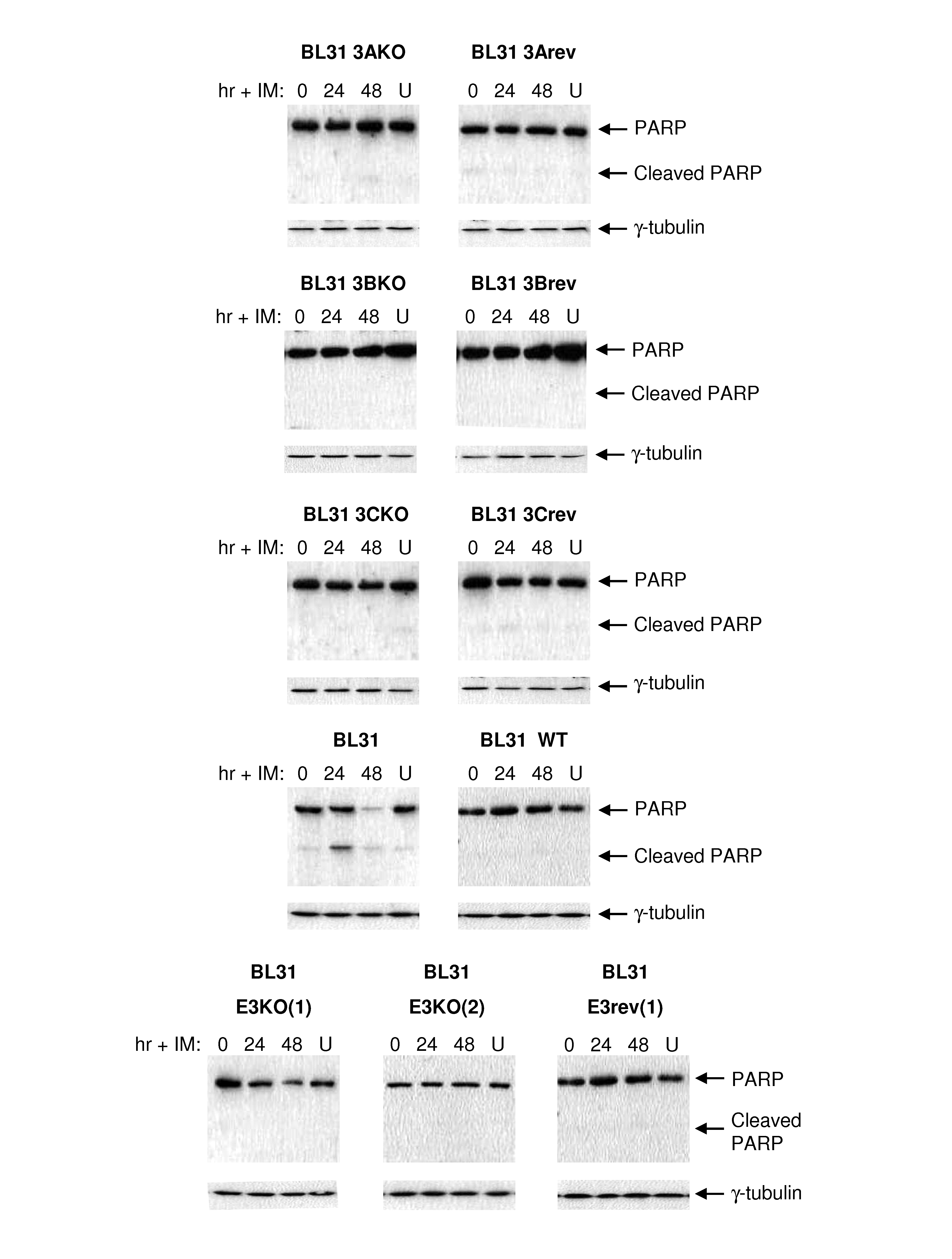

Supplement: Figure S2 — The EBNA3 locus is not involved in the EBV-mediated protection against ionomycin-induced apoptosis. The EBV-negative BL31 cell line, its recombinant B95.8-BAC EBV convert, BL31 cell lines established using individual EBNA3A-, 3B- and 3C-knockout viruses and their respective revertants, as well as BL31 cells lines converted with recombinant EBNA3 locus-knockout (E3KO) and revertant (E3rev) EBVs were all treated with 1 µg/ml ionomycin (IM) for up to 48 hours. Protein was extracted from cells harvested at the start of the treatment and at the times indicated. Protein extracts were separated by SDS-PAGE and analysed by western blotting using antibodies that detect full-length and cleaved PARP. U indicates untreated control cells after 48 hours. Throughout, γ-tubulin was used as a loading control. (TIF) [file pone.0028506.s002.tif]

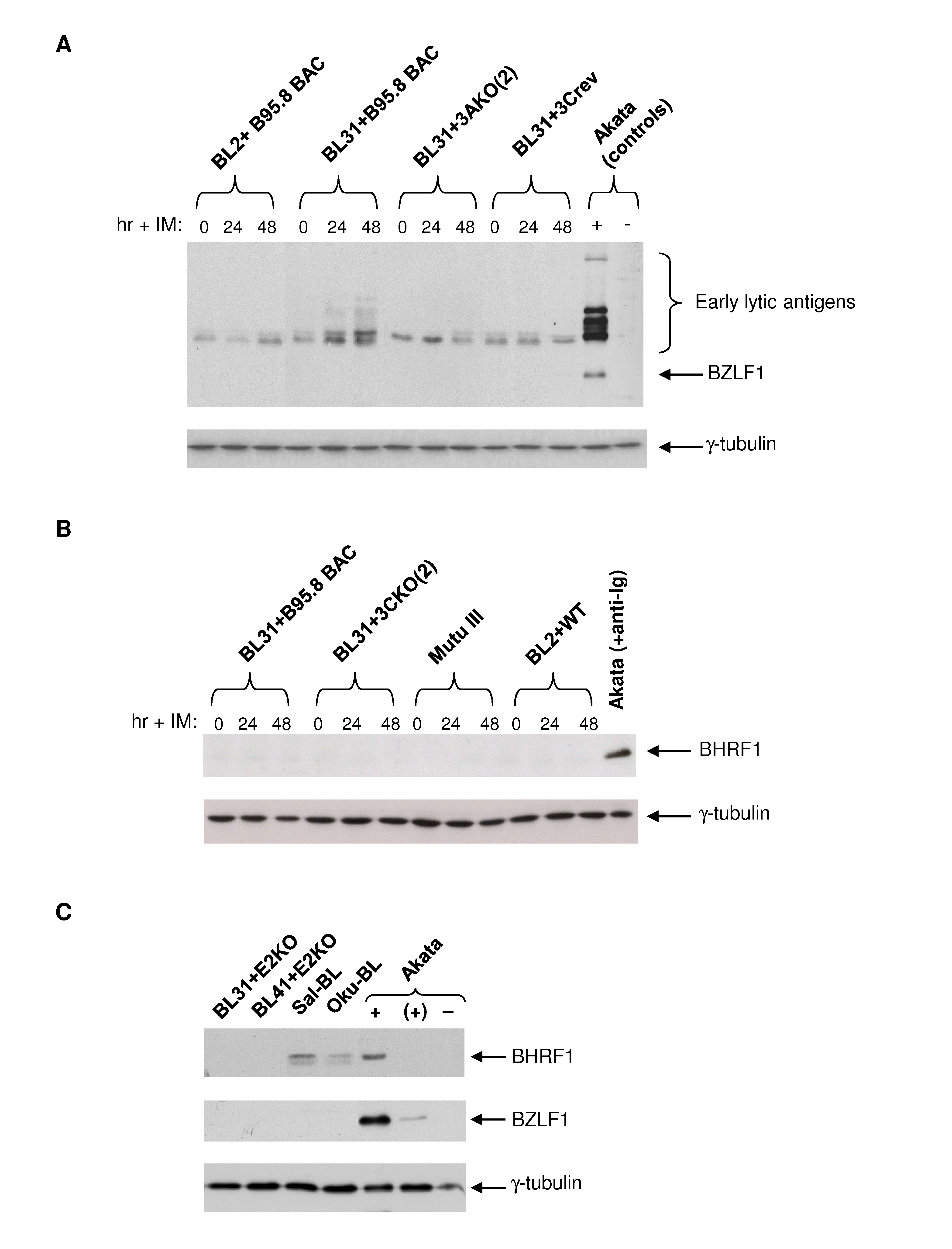

Supplement: Figure S3 — Rescue from apoptosis induced by ionomycin is not dependent on EBV lytic gene expression and Wp-restricted BL lines express BHRF1. Various EBV-positive BL cells were treated with 1 µg/ml ionomycin (IM) for 48 hours and harvested at the times indicated. Protein extracts from treated cells were separated by SDS-PAGE and analysed by western blotting using (A) the human serum EE which detects lytic antigens and (B) and (C) monoclonal antibodies against BHRF1 and BZLF1. Extracts from Akata cells [47] left untreated (−) or treated with anti-Ig [+ or (+)] to stimulate the expression of EBV lytic proteins were included to show lytic gene expression. BZLF1 was used as a control for lytic activation and γ-tubulin was used a loading control. (TIF) [file pone.0028506.s003.tif]

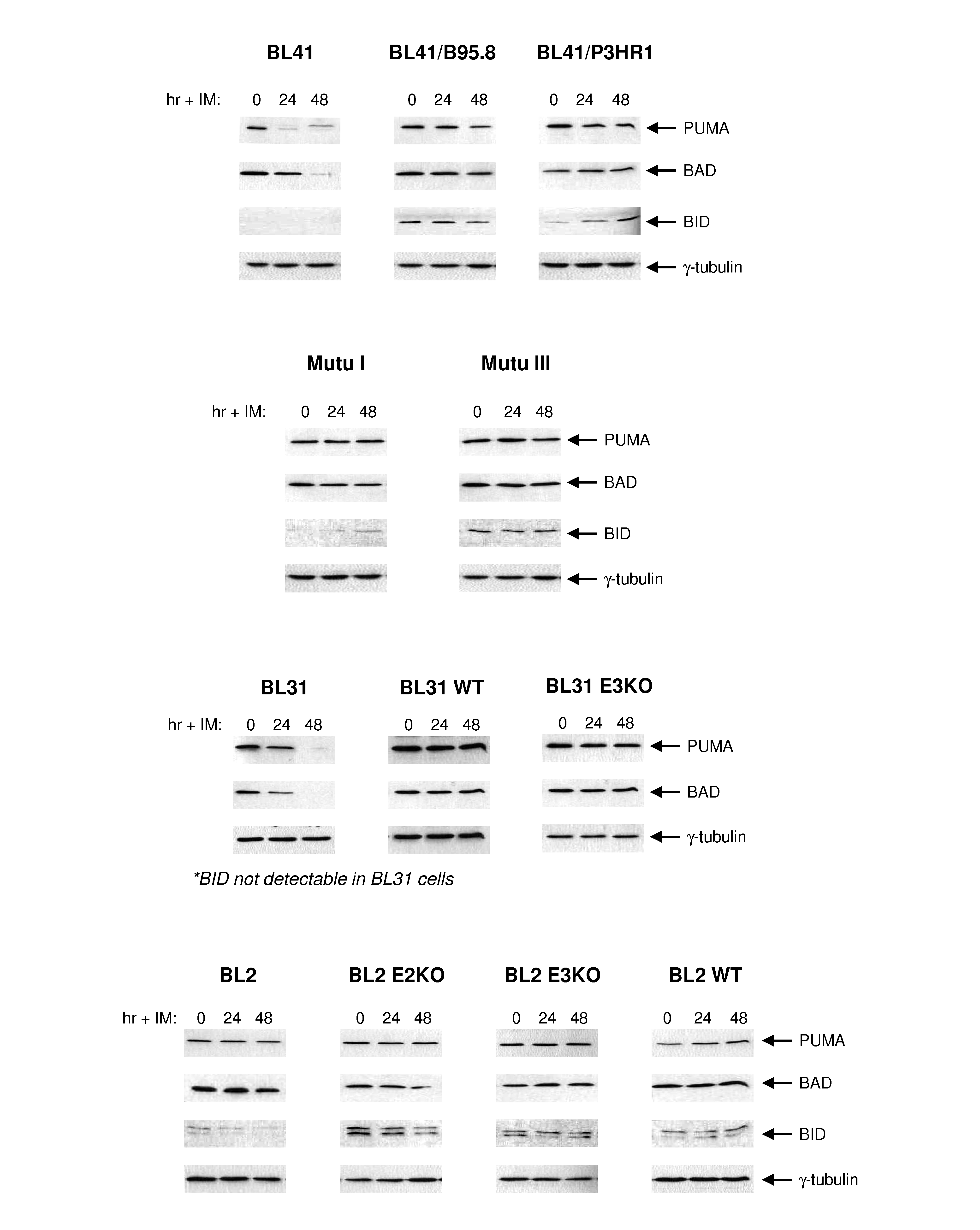

Supplement: Figure S4 — Ionomycin does not consistently induce expression of BAD, BID or PUMA. Protein extracts from representative BL-derived cells treated with 1 µg/ml ionomycin (IM) for up to 48 hours were analysed by western blotting using antibodies directed against the pro-apoptotic BH3-only factors PUMA, BAD and BID. Throughout, γ-tubulin was used as a loading control. (TIF) [file pone.0028506.s004.tif]

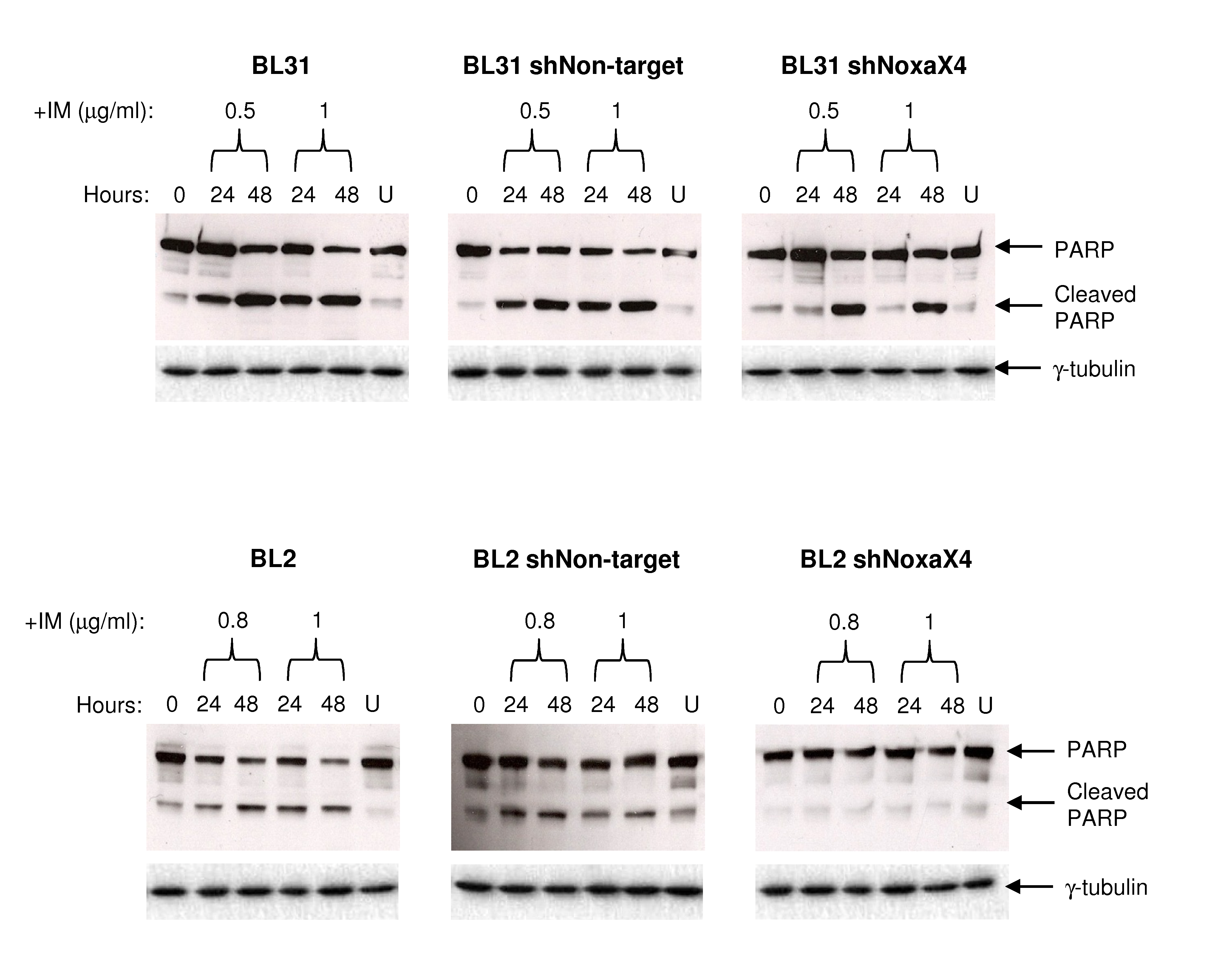

Supplement: Figure S5 — shRNA-mediated knockdown of NOXA increases resistance to ionomycin-induced apoptosis. BL31 and BL2 cells established using lentiviral vectors expressing shRNA targeted against NOXA were exposed to ionomycin (IM) at the concentrations indicated for up to 48 hours. BL cells established using lentiviruses expressing a non-targeting shRNA were included as controls. Protein was extracted from cells harvested at the start of the treatment and the times indicated. Western blotting was performed for evidence of PARP cleavage. U indicates vehicle-treated control cells after 48 hours. Throughout, γ-tubulin was used as a loading control. (TIF) [file pone.0028506.s005.tif]

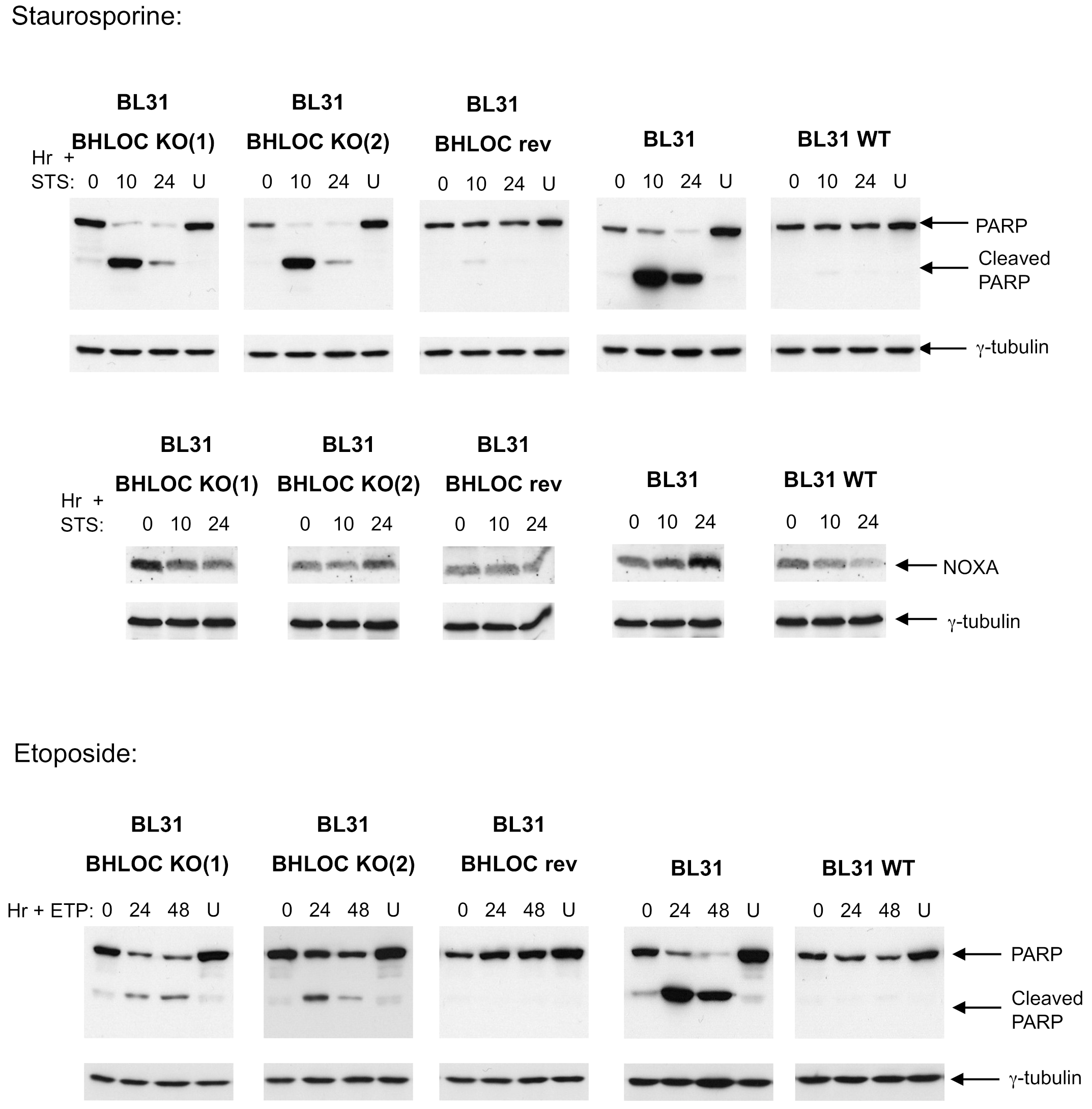

Supplement: Figure S6 — The BHRF1 locus contributes to the EBV-mediated protection against apoptosis induced by both staurosporine and etoposide. EBV-negative BL31, its recombinant B95.8-BAC EBV convert and BL31 cell lines established with recombinant BHRF1 locus-knockout (BHLOC KO) and revertant (BHLOC rev) EBVs were treated with 0.25 µM staurosporine (STS) for up to 24 hours or 500 ng/ml etoposide (ETP) for up to 48 hours. Protein was extracted from cells harvested at the start of the treatment and the time points indicated. Western blotting was performed using antibodies directed against PARP (top and bottom panels) and directed against NOXA (middle panel). U indicates vehicle-treated control cells after 24 or 48 hours. Throughout, γ-tubulin was used as a loading control. (TIF) [file pone.0028506.s006.tif]

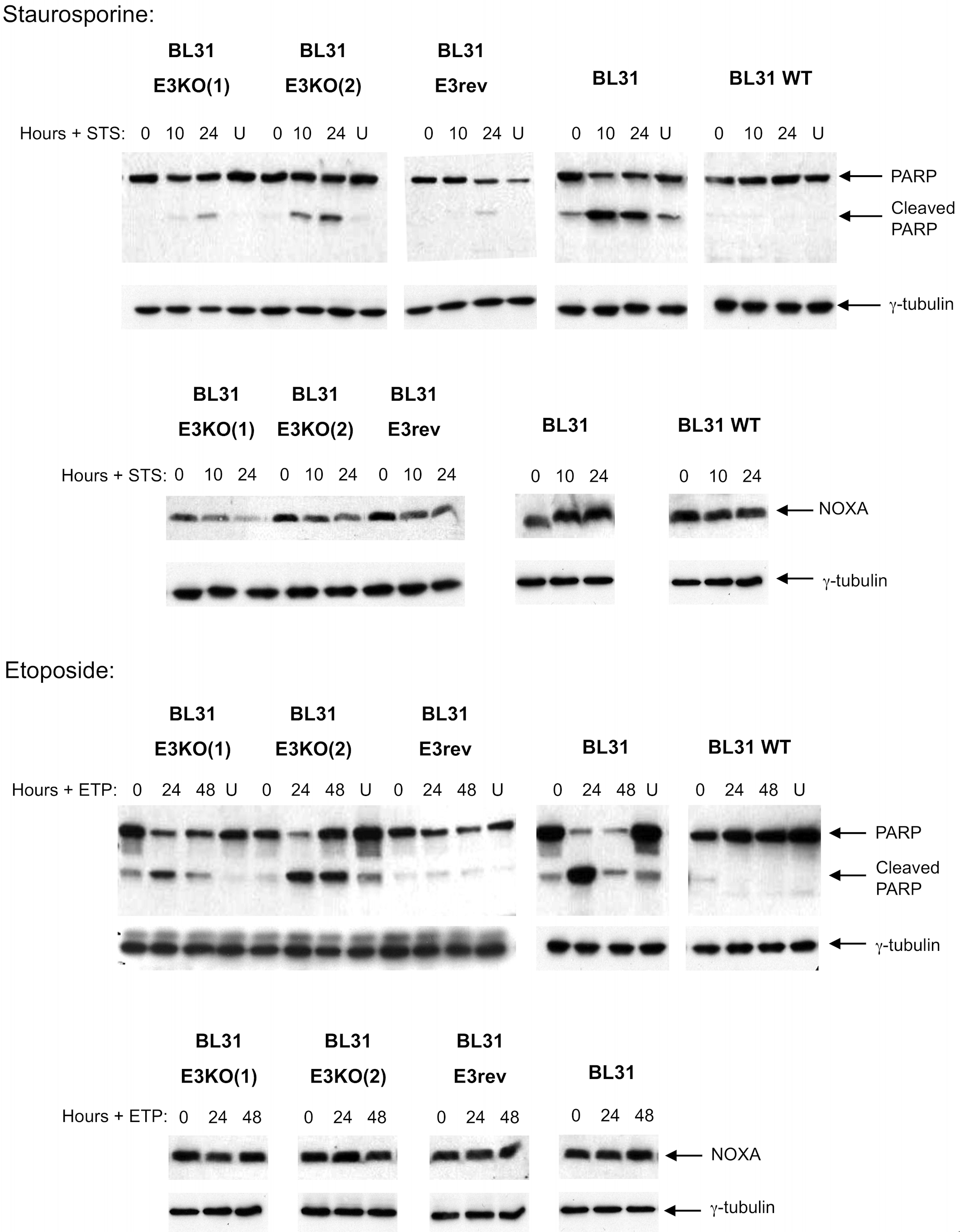

Supplement: Figure S7 — The EBNA3 locus protects against etoposide (genotoxin)-, but not staurosporine-induced apoptosis. Similar experiments and analysis to (S6) were performed using BL31 cell lines established using recombinant EBNA3 locus-knockout (E3KO) and revertant (E3rev) EBVs. (TIF) [file pone.0028506.s007.tif]
